# Membrane dynamics of resting and internalin B‐bound MET receptor tyrosine kinase studied by single‐molecule tracking

**DOI:** 10.1002/2211-5463.12285

**Published:** 2017-08-29

**Authors:** Marie‐Lena I. E. Harwardt, Phoebe Young, Willem M. Bleymüller, Timo Meyer, Christos Karathanasis, Hartmut H. Niemann, Mike Heilemann, Marina S. Dietz

**Affiliations:** ^1^ Institute of Physical and Theoretical Chemistry Johann Wolfgang Goethe‐University Frankfurt Germany; ^2^ Structural Biochemistry Department of Chemistry Bielefeld University Germany

**Keywords:** diffusion dynamics, endocytosis, internalin B, MET receptor, receptor tyrosine kinases, single‐molecule tracking

## Abstract

The human MET receptor tyrosine kinase contributes to vertebrate development and cell proliferation. As a proto‐oncogene, it is a target in cancer therapies. MET is also relevant for bacterial infection by *Listeria monocytogenes* and is activated by the bacterial protein internalin B. The processes of ligand binding, receptor activation, and the diffusion behavior of MET within the plasma membrane as well as its interconnections with various cell components are not fully understood. We investigated the receptor diffusion dynamics using single‐particle tracking and imaging fluorescence correlation spectroscopy and elucidated mobility states of resting and internalin B‐bound MET. We show that internalin B‐bound MET exhibits lower diffusion coefficients and diffuses in a more confined area in the membrane. We report that the fraction of immobile receptors is larger for internalin B‐bound receptors than for resting MET. Results of single‐particle tracking in cells treated with various cytotoxins depleting cholesterol from the membrane and disrupting the actin cytoskeleton and microtubules suggest that cholesterol and actin influence MET diffusion dynamics, while microtubules do not have any effect.

**Abbreviations**
EGFR: epidermal growth factor receptor
Gab1: Grb2‐associated bind protein 1
Grb2: growth factor receptor‐bound protein 2
HGF/SF: hepatocyte growth factor/scatter factor
imFCS: imaging fluorescence correlation spectroscopy
InlB: internalin B
MCD: methyl‐β‐cyclodextrin
MSD: mean square displacement
NA: numerical aperture
PLL‐PEG: poly‐L‐lysine‐grafted polyethylene glycol
ROI: region of interest
RTK: receptor tyrosine kinases
TIRF: total internal reflection fluorescence
TrkA: neurotrophin receptor tropomyosin‐related kinase A
uPAINT: universal point accumulation for imaging in nanoscale topography

Receptor tyrosine kinases (RTKs) are transmembrane receptors which are bound and activated by a variety of ligands, including growth factors, differentiation factors, and hormones 1. RTKs represent the primal elements of several cellular signaling cascades and exhibit distinct regulative effects on cellular proliferation, differentiation, metabolism, motility, and cell‐to‐cell signaling. As receptor dynamics and interactions are involved in a series of cellular processes, research on this topic is particularly worthwhile. Mutations in RTK‐encoding gene sequences may result in altered functions or structures, while alterations in regulatory domains cause receptor overexpression, all of which are thought to trigger or promote various diseases such as different types of cancer, inflammation, and diabetes. This renders RTKs important targets for many pharmaceutical therapies 1, 2.

MET belongs to the RTK family and is found in various eukaryotic cells including those of humans. Upon activation by its native ligand, hepatocyte growth factor/scatter factor (HGF/SF), the MET receptor triggers intracellular signaling cascades which result in various cellular responses such as cell motility and growth 3. MET signaling is attenuated by receptor internalization and degradation 4, 5.

MET is also targeted by *Listeria monocytogenes*. This pathogenic bacterium can mediate a bacterial infection of the central nervous system through its surface proteins internalin (InlA) and internalin B (InlB) 6. InlB activates MET and initiates signaling pathways and endocytosis analog to HGF/SF 6, 7. Internalization of active InlB/MET complexes is mediated by clathrin‐coated pits 8, while lipid domains in the cell membrane are crucial for uptake of *L. monocytogenes* into host cells 9. It was shown that a fragment of InlB comprising amino acids 36–321, InlB_321_, is sufficient to bind and activate MET 10. Previous single‐molecule studies used InlB_321_ binding to investigate the oligomeric state of MET. It was found that the resting MET receptor is present as monomer and dimer and that binding of InlB_321_ increases the fraction of dimers 11.

Functional properties of proteins in the plasma membrane are regulated by its lipid and protein composition and by interactions between biomolecules within the membrane as well as with intracellular proteins 12. A variety of drugs are available which influence specific components of this complex network 13 and aid in identifying those interactions that are relevant for a particular membrane protein. In terms of lipids, cholesterol is an important component of the membrane affecting its structure, fluidity, and functionality as well as several processes occurring at the membrane 14. Methyl‐β‐cyclodextrin (MCD) encapsulates cholesterol and swiftly depletes it from cell membranes 15. Additionally, it mainly inhibits clathrin‐mediated endocytosis by preventing the invagination and pinching‐off of clathrin‐coated pits, resulting in numerous flat, clathrin‐coated pits on the cell surface. It also affects caveolae‐dependent endocytosis pathways 16, 17. Nystatin is able to chelate and deplete cholesterol. It inhibits caveolae‐dependent endocytosis by flattening caveolae at the cell surface 18.

The plasma membrane is further organized by cytoskeletal proteins. Actin filaments are crucial components of the cytoskeleton, organize the cytoplasm, and determine cell shape. The dynamic polymerization of actin and subsequent reshaping of the cell membrane orchestrate cell migration and endocytosis 19. Actin partitions the membrane into domains and provides anchoring sites for membrane proteins 20. Cytochalasin D is able to bind actin, preventing association of further actin monomers, rupturing already existing actin polymers, and disrupting the supramolecular organization of the actin cytoskeleton 21. Latrunculin A binds to actin monomers, inhibiting further actin polymerization and depolymerizing actin filaments 22. Microtubules are another component of the cytoskeleton. They are responsible for cell shape and play a role in cellular transport 23. Colchicine binds to tubulin, preventing assembly into microtubules. Depolymerization of microtubules accelerates with increasing colchicine concentration as lateral contacts between tubulin molecules attenuate 24.

Manipulating cells with cytotoxins allows the identification of molecular interactions and mechanisms. Sensitive microscopy and spectroscopy methods, such as single‐molecule tracking or fluorescence correlation spectroscopy, read out diffusion dynamics of membrane receptors in live cells and report on diffusion types and diffusion coefficients. These observables are sensitive to subtle alterations of protein dynamics within the membrane and, if investigated under different cellular conditions, allow the deduction of mechanistic aspects of receptor activation and internalization.

In this study, we investigated the diffusion of resting and InlB‐bound MET within the plasma membrane of living cells using single‐particle tracking following the uPAINT protocol 25 and imaging fluorescence correlation spectroscopy (imFCS) 26, 27. Both methods apply TIRF illumination, which allows selective excitation of labeled ligands bound to receptors at the basal cell membrane under exclusion of background fluorescence from regions deeper in the cell. We investigated how MET diffusion is influenced if cells are treated with cytotoxins that deplete cholesterol or affect actin polymerization. We determined diffusion coefficients and identified populations of diffusion types of single MET receptors by analyzing trajectories using fluorophore‐labeled ligands. We found that InlB‐bound MET exhibits lower average diffusion coefficients than resting MET. We show that the perturbation of actin polymerization resulted in increased diffusion coefficients for InlB‐bound MET, whereas resting MET was not affected. Treatment with MCD, which inhibits clathrin‐mediated endocytosis but may also affect caveolae‐dependent internalization, resulted in distinctly decreased diffusion coefficients for InlB‐bound MET and slightly lower diffusion coefficients for resting MET; treatment with nystatin, which inhibits caveolae‐dependent endocytosis, had a small effect on the diffusion of resting MET, but not on diffusion of the InlB/MET complex.

## Materials and methods

### Coverslip passivation and functionalization

25‐mm borosilicate glass coverslips (VWR International, Radnor, PA, USA) were passivated with poly‐L‐lysine‐grafted polyethylene glycol (PLL‐PEG) to minimize unspecific interactions with the glass surface and partly functionalized for cell adhesion with a peptide (CG**RGD**S) containing the motif arginyl‐glycyl‐aspartic acid (RGD) 28. PLL‐PEG‐RGD was a kind gift of Jacob Piehler (Osnabrück University). Coverslips were sonicated (Sonorex SUPER RK 102H, Bandelin, Berlin, Germany) in isopropyl alcohol (Sigma‐Aldrich, St. Louis, MO, USA) for 20 min at 23 °C, rinsed with bidistilled water (Arium Pro Ultrapure Water Systems, Sartorius, Goettingen, Germany), dried with nitrogen, and plasma cleaned for 10 min at 80% power and 0.2–0.3 mbar (Zepto B; Diener Electronic GmbH, Ebhausen, Germany). Ten microlitre of 0.8 mg·mL^−1^ PLL‐PEG‐RGD solution in 1 × phosphate‐buffered saline (PBS) pH 7.4 (prepared from 10 × concentrate, catalog# D1408; Sigma‐Aldrich) was incubated between two coverslips for approximately 90 min at 23 °C. Coverslips were separated by rinsing with bidistilled water, dried with nitrogen, and stored at −20 °C until further use in six‐well plates (Greiner Bio‐One International GmbH, Frickenhausen, Germany) covered with argon.

### Cell culture

All experiments were performed in HeLa cells (Institut für angewandte Zellkultur, Munich, Germany). Cells were cultivated in growth medium (GM) consisting of high glucose Dulbecco's modified Eagle medium (DMEM) (Gibco, Life Technologies, Waltham, MA, USA) with 1% GlutaMAX (Gibco, Life Technologies) and 10% fetal bovine serum (Gibco, Life Technologies) in cell culture flasks (250 mL, 75 cm^2^; Greiner Bio‐One, Kremsmuenster, Austria) at 37 °C and 5% CO_2_ in an automatic CO_2_ incubator (Model C 150; Binder GmbH, Tuttlingen, Germany) for three to four days. When a cell passage needed to be split, GM was removed from the flask. After cells were rinsed with Dulbecco's PBS (Gibco, Life Technologies), 2 mL trypsin solution (0.05% trypsin, 0.02% EDTA; Invitrogen, Waltham, MA, USA) was added and incubated for 3 min at 37 °C to detach cells. Cells were suspended in 10 mL GM and centrifuged at 300 ***g*** for 3 min (Multifuge X1R; Thermo Scientific, Waltham, MA, USA). Cells were resuspended in GM, and a fraction was filled into a new cell culture flask with 15 mL fresh GM.

For experiments, HeLa cells were seeded onto PLL‐PEG‐RGD‐coated coverslips in six‐well plates. Each well was filled with 2 mL GM containing 5 × 10^4^ cells. Subsequently, the well plate was incubated at 37 °C and 5% CO_2_ for 3 days. Prior to measurements, coverslips were transferred to custom‐made coverslip holders. According to the intended measurements, either solution of cytotoxins in GM or pure GM was added to the cells in the coverslip holder.

### uPAINT

#### Sample preparation: fluorescent labeling of ligands

InlB_321_ was expressed with a lysine mutated into a cysteine (K280C) and a native cysteine mutated into an alanine (C242A) in order to create a single site for maleimide coupling and at the same time prevent the formation of intramolecular disulfide bonds. These mutations do not impair MET binding 10, 29. The thiol group of the mutated cysteine was coupled to ATTO 647N maleimide (ATTO‐TEC, Siegen, Germany), and labeled InlB_321_ was purified according to Dietz *et al*. 11.

The antibody fragment 3H3‐Fab (produced from a high‐affinity, monoclonal antibody against the MET ectodomain) is able to bind but not activate MET. Fab was labeled by coupling ATTO 647N‐*N*‐hydroxysuccinimide ester (ATTO‐TEC) with lysines of the antibody fragment. 2.45 nmol of Fab and 9.8 nmol of ATTO 647N‐NHS ester (4 × dye excess) were dissolved in 100 μL of PBS with 20 mm NaHCO_3_ (pH 8) (Sigma‐Aldrich). The mixture was slightly shaken under exclusion of light for 2 h at 23 °C. Labeled Fab fragment was separated from unbound dye molecules by chromatography with a NAP‐5 column (GE Healthcare, Dornstadt, Germany), equilibrated with 10 mL PBS, and eluted with PBS. The concentration of the labeled ligand and the degree of labeling (DOL) were determined by absorption spectroscopy (Nanophotometer; Implen GmbH, Munich, Germany). A yield of 26% and a DOL of 164% were obtained. Both ligands were stored at −20 °C in protein LoBind tubes (Eppendorf GmbH, Hamburg, Germany).

#### Sample preparation: staining for uPAINT

uPAINT measurements were taken at 23 °C and at 32 °C. Approaches for the respective conditions differ slightly. For measurements at 23 °C, 600 μL of GM was pipetted onto the cells in the coverslip holders, and left to gradually cool to 23 °C for 15 min. Then, the coverslip was rinsed once with 600 μL of serum‐free imaging medium (IM) containing high glucose DMEM with 1% GlutaMAX and 50 mM HEPES buffer (pH 7.2–7.5; Gibco, Life Technologies). Fresh solutions of both labeled ligands, InlB_321_ and Fab, were prepared with concentrations of 50 nm in IM. Three microlitre of the respective solution and 597 μL of IM were mixed on the coverslip resulting in a final ligand concentration of 0.25 nm.

For measurements at 32 °C, the glass coverslip positioned in the coverslip holder was rinsed once with warm IM. 599 μL of IM was added and the coverslip was positioned onto the objective of the microscope, which was heated to 37 °C and allowed to acclimate for 10 min. The temperature in the sample itself was 32 °C. 1.2 μL of the respective fresh 50 nm ligand solution in IM was added to the solution in the coverslip holder (final concentration of ligand 0.1 nm) without removing the sample from the objective. For both temperature settings, a measurement was started approximately three minutes after the addition of a fluorescently labeled ligand so that the ratio of bound to unbound receptors was already balanced.

#### Sample preparation: exposure to cytotoxins

All cytotoxins were ordered from Sigma‐Aldrich. Stock solutions of nystatin, cytochalasin D, latrunculin A, and colchicine were prepared in dimethyl sulfoxide, while MCD was dissolved in bidistilled water. All solutions were stored at −20 °C.

Cells exposed to 600 μL of MCD (10 mm), nystatin (50 μm), cytochalasin D (1 μm), or colchicine (10 μm) in GM were incubated for 15 min at 37 °C, while cells exposed to latrunculin A (1 μm) were incubated for 10 min. Following incubation, coverslips were rinsed with 600 μL of GM. Ligand addition corresponded with the protocol described in the previous section. uPAINT measurements of cells exposed to cytotoxins were performed at 23 °C for each ligand.

#### Setup and data acquisition

Data were collected with a N‐STORM microscope (Nikon, Duesseldorf, Germany) with a 647‐nm laser for ATTO 647N excitation, applying total internal reflection fluorescence (TIRF) illumination. Measurements were started and controlled by the application of two software tools: Micro‐Manager 30 and NIS Elements (Nikon). The objective (100 × Apo TIRF oil) had a 100 × magnification and a numerical aperture (NA) of 1.49 and could be adjusted to temperatures of 23 and 37 °C as well as to the thickness of the coverslips (0.17 mm). The frame transfer of the camera (DU‐897U‐CS0‐BV; Andor Technology, Belfast, UK) was used. A laser intensity of 0.1 kW·cm^−2^ and an EM gain of 200 were employed. The image size was limited to 256 × 256 pixels with a pixel size of 0.158 μm. Films were recorded with 1000 frames and an exposure and frame time of 20 ms. The laser was switched on only during measurements to minimize phototoxicity. On each coverslip, measurements were taken on 20 living cells, never exceeding an overall time period of 30 min per sample. Ten films of background were recorded on positions devoid of cells.

#### Image analysis

Films were analyzed using PALM‐Tracer (Bordeaux Imaging Center), which is a plugin for MetaMorph (Molecular Devices, Sunnyvale, CA, USA). The following settings were chosen: mean square displacement (MSD) fitting length was four points, and the maximum distance between two consecutive localizations was set at five5 pixels (790 nm); that is, points further apart were not connected to a trajectory. Only trajectories with a minimum length of 20 frames were accepted. The threshold was set according to the value with the lowest background signal within the last frame. Regions of interest (ROI) were set by manually outlining the shape of cells in the transmitted light images and subsequently transferring these outlines to the respective films. Background films were analyzed without the application of a ROI. The software finds localization points, fits them with a centroid function, and connects them according to the set criteria. Analysis yielded x‐y‐coordinates of each localization, MSD values, and diffusion coefficients for individual trajectories obtained by fitting the first four points of the MSD plots with Eqn (1).

(1) MSD(Δt)=4DΔt.

#### Data analysis: generation of logarithmic diffusion coefficient histograms

Sixty cells from at least two different days were selected for each treatment group. Diffusion coefficients for each trajectory of these cells were imported into Origin (OriginPro 2016G; OriginLab Corporation, Northampton, MA, USA) as well as the diffusion coefficients determined in background measurements. Data were log‐transformed and binned in the range between −5.3 and 1.0 with a bin size of 0.1 for each measurement. All frequency counts were normalized to 1 μm^2^. Frequency counts for the background measurements of one coverslip were averaged and subtracted from the respective frequency counts of each cell on the same coverslip. Frequency counts of background‐corrected cells were averaged over all selected 60 cells and normalized. Logarithmic diffusion coefficient values were re‐transformed. Frequency counts were plotted logarithmically against diffusion coefficients.

#### Data analysis: dynamic localization precision

The localization precision of dynamic particles in the recorded films was determined according to the method described by Michalet 31. The y‐intercept of the MSD plot MSD (0), the diffusion coefficient *D*, and the frame time 0.02 s influence the dynamic localization precision σ_dyn_ (Eqn (2)).

(2) $$\sigma_{\mathrm{dyn}}=\sqrt{\frac{\text{MSD}\left(0\right)+\frac{4}{3}D\cdot 0.02s}{4}}.$$

Nine cells from three different days were selected for either InlB_321_ or Fab. Diffusion coefficients and MSD (0) values were averaged per cell. σ_dyn_ was calculated for all nine cells. The third quartile of these nine values was adopted as dynamic localization precision which amounted to 45.5E‐3 μm for InlB_321_ ATTO 647N in IM. The same value was applied to trajectories of Fab‐ATTO 647N in order to guarantee comparability of diffusion types for all samples.

#### Data analysis: assignment of diffusion types

Diffusion coefficients were assigned to three types of diffusion: immobile, confined, and free. The dynamic localization precision and the method described by Rossier *et al*. were used for the assignment 32. Particles were defined as immobile if their diffusion coefficient was lower than the lowest determinable value *D*_min_ on the basis of the dynamic localization precision. *D*_min_ was determined by rearranging Eqn (1) to calculate *D*. MSD(Δ*t*) was substituted with $\sigma_{\mathrm{dyn}}^{2}$ , and (Δ*t*) corresponds to the time period of the first four MSD points 4·0.02 s as only these were fitted to determine the diffusion coefficient (Eqn (3)).

(3) $$D_{\min}=\frac{\sigma_{\mathrm{dyn}}^{2}}{4\cdot 4\cdot 0.02\;s}.$$

According to this equation, the *D*_min_ value for InlB_321_‐ATTO 647N in IM amounted to 6.5E‐3 μm^2^·s^−1^; that is, all particles with a diffusion coefficient smaller than this value were assigned as immobile. This value was applied for all treatment groups to ensure a comparable classification of diffusion. Further differentiation between confined and free diffusion was performed according to Rossier *et al*. 32.

#### Data analysis: cumulative analysis of diffusion coefficients

A second method to deduce diffusion types is a cumulative analysis according to the equations proposed by Gebhardt *et al*. 33. Cumulative frequencies were calculated for the logarithmic diffusion coefficients in an interval between −5.3 and 1.4 for each cell. These frequencies were averaged over 60 cells per treatment group and normalized. The logarithmic values were re‐transformed, and the cumulative frequencies were plotted against diffusion coefficient values on a logarithmic scale. These plots were fitted with an equation for one population (Eqn (4)) and for two populations (Eqn (5)).

(4) $$F\left(D\right)=A_{1}\left(1-\text{exp}\left(-\frac{D}{D_{1}}\right)\right),$$

(5) $$\begin{array}{cc} F(D)=\; & A_{1}\left(1-\text{exp}\left(-\frac{D}{D_{1}}\right)\right)\\ & +\left(1-A_{1}\right)\left(1-\text{exp}\left(-\frac{D}{D_{2}}\right)\right). \end{array}$$

*A*_1_ represents the amplitude of the cumulative fit. In the case of two populations, it equals the fraction of molecules belonging to population 1, while (1 − *A*_1_) represents the fraction of particles belonging to population 2. Residual curves were calculated and plotted to give an impression of the quality of the fits.

### imFCS

#### Sample preparation

Staining for imFCS experiments was conducted in an analog fashion to uPAINT experiments. However, higher final ligand concentrations of 50 nm in IM needed to be applied to obtain sufficient signal to calculate autocorrelation curves. Samples were not exposed to any drugs.

#### Setup and data acquisition

The experimental setup for imaging FCS measurements used a 640‐nm diode laser (100 mW, Obis; Coherent, Santa Clara, CA, USA) as an excitation source passing through a telescope consisting of two achromatic lenses (Thorlabs, Munich, Germany) with *f* = −40 mm and 750 mm. A third achromatic lens (*f* = 400 mm; Thorlabs) directed the excitation light to the TIRF mirror and had its focus on the back focal plane of the objective. The position of the TIRF mirror was changed to switch between wide‐field and TIR illumination. The light entered an Eclipse Ti microscope (Nikon), was reflected by a dichroic mirror (zt532/640rpc; AHF, Tuebingen, Germany), and was directed onto the sample by an oil immersion TIRF objective (100 ×, NA 1.45; Nikon). Emission light was collected by the same objective, passed the dichroic mirror, and was detected by a scientific complementary metal oxide semiconductor (sCMOS) camera (Zyla 4.2; Andor Technology, South Windsor, CT, USA). The open‐source software Micro‐Manager was used for data collection. For data acquisition, the following settings were applied: 0.03 kW·cm^−2^ laser intensity (640 nm), a bit depth of 16 bit, fastest readout, exposure time 1 ms, frame time 1 ms, 4 × 4 binning, and 50 000 frames per film. Each film contained a 40 × 20 pixel ROI. On each coverslip, measurements were taken on up to 13 living cells, never exceeding an overall time period of 30 min per sample.

#### Image analysis

Analysis of imFCS films was performed with Imaging_FCS 1.47 34, which is a plugin for ImageJ (NIH, Bethesda, MD, USA) 35. The following correlation settings were chosen: emission wavelength 669 nm for ATTO 647N, NA of 1.45, correlator scheme *P* = 16 and *Q* = 8, lateral PSF = 0.90, binning = 1, magnification 25 for 4 × 4 binning, and linear segment bleach correction with step width 5000. PSF calibration was performed according to Bag *et al*. 36. Diffusion coefficients were directly obtained for each pixel by fitting the correlation curves according to the literature 34.

#### Generation of logarithmic diffusion coefficient histograms

Sixty cells from three different days were selected for each ligand. Diffusion coefficients were imported into Origin. Data were log‐transformed and binned in the interval between −5.3 and 1.0 with a bin size of 0.1 for each cell. Frequency counts were averaged over all selected 60 cells and normalized. Logarithmic diffusion coefficients were re‐transformed. Frequency counts were plotted logarithmically against diffusion coefficients.

### Statistics

If not stated otherwise, mean values are listed with respective standard errors of the mean (SEM, Δ). Average values and SEMs were determined for each cell. Overall means were calculated by averaging the mean values of 60 cells (Eqn (6)). The overall SEMs were determined according to Gaussian error propagation by averaging the SEMs of all 60 cells (Eqn (7)).

(6) $$D_{\mathrm{mean}}=\frac{1}{60}\sum_{i=1}^{60}D_{i}.$$

(7) $$\Delta D_{\mathrm{mean}}=\frac{1}{60}\sum_{i=1}^{60}\frac{\delta D_{\mathrm{mean}}}{\delta D_{i}}\cdot \Delta D_{i}=\frac{1}{60}\sum_{i=1}^{60}\Delta D_{i}.$$

*t*‐Tests were used to validate the comparison of mean values. Paired *t*‐tests (α = 0.05) were applied to compare means obtained by observations of the same cells, for example, free and confined diffusion coefficients of one treatment group. Two‐sample *t*‐tests (α = 0.05) were used to validate statements considering means of two different treatment groups, for example, the difference in diffusion coefficients of untreated cells compared to the diffusion coefficients of cells treated with a cytotoxin. Levels of significance were classified as follows: *P* > 0.05 no significant difference between means (n.s.), *P* < 0.05 significant difference (*), *P* < 0.01 very significant difference (**),*P* < 0.001 highly significant difference (***). Datasets of all treatment groups were tested for normality by applying the Kolmogorov–Smirnov test (α = 0.1). All tests were performed in Origin.

### Confocal laser scanning microscopy of cytotoxin‐treated cells

#### Sample preparation

Exposure to cytotoxins was performed on living cells analog to the uPAINT experiments. Subsequently, cells were incubated in fresh GM at 23 °C for 15 min. These time intervals correspond to the periods of incubation before a uPAINT measurement. Control samples were prepared which were not exposed to any cytotoxin prior to staining and fixation.

#### Actin staining

After the removal of GM, cells were fixed by adding 600 μL of 4% (v/v) formaldehyde (Sigma‐Aldrich) in PBS and incubating for 10 min. Cells were rinsed three times with 600 μL of PBS and permeabilized with 600 μL of 0.1% (v/v) Triton X‐100 (TX) (Sigma‐Aldrich) in PBS for 10 min. Actin was stained with 600 μL of 13.2 nm phalloidin‐Alexa Fluor 647 (New England Biolabs, Hitchin, UK) in PBS for 10 min. Stained cells were rinsed with 600 μL of PBS. If not imaged immediately, coverslips were stored in PBS containing 0.05% (w/v) NaN_3_ (Roth, Karlsruhe, Germany) at 4 °C.

#### Microtubules staining

Cells were incubated in 600 μL of microtubule‐stabilizing buffer containing 80 mm PIPES (Sigma‐Aldrich), 1 mm MgCl_2_ (Sigma‐Aldrich), 5 mm EGTA (Sigma‐Aldrich), and 0.5% (v/v) TX. After 30 s, 0.5% (w/v) glutaraldehyde (Sigma‐Aldrich) was added and incubated for 10 min. The latter was quenched by replacement with approximately 0.1% (w/v) NaBH_4_ (Sigma‐Aldrich) in PBS for 7 min. Subsequently, fixed cells were rinsed three times with 600 μL of PBS and incubated for 10 min in antibody‐diluting buffer (AbDil) consisting of 0.1% (v/v) TX and 2% (w/v) bovine serum albumin (Sigma‐Aldrich) in PBS. Cells were exposed to 600 μL of primary antibody solution containing 1 μg·mL^−1^ monoclonal mouse anti‐β‐tubulin IgG1 (Life Technologies, USA, catalog# 32‐2600) in AbDil for 30 min. After rinsing four times with 0.1% (v/v) TX in PBS, incubation with donkey anti‐mouse IgG‐Alexa Fluor 647 (1 μg·mL^−1^ in AbDil) (Life Technologies, USA) was performed for 30 min. Then, cells were rinsed four times with PBS containing 0.1% TX and a further four times with pure PBS. A postfixation step was performed by adding 600 μL of 4% (v/v) formaldehyde for 10 min. Finally, cells were rinsed four times with pure PBS. If not imaged immediately, cells were stored in PBS containing 0.05% (w/v) NaN_3_ at 4 °C.

#### Staining with fluorescently labeled cholera toxin B

For staining of cholesterol‐rich regions, living cells were incubated with the respective cytotoxins together with 2 μg·mL^−1^ cholera toxin subunit B‐Alexa Fluor 647 (Life Technologies, USA) in GM for 15 min at 37 °C. Cholera toxin subunit B is known to bind to ganglioside monosialotetrahexosylganglioside (GM1) which is localized in cholesterol‐rich nanodomains 37. After rinsing with 600 μL of GM, cells were incubated for 15 min at 23 °C. Samples were fixed by the addition of 600 μL of 4% (v/v) formaldehyde in PBS and incubation for 10 min. Once rinsed three times with PBS, cells were imaged or stored in PBS containing 0.05% (w/v) NaN_3_ at 4 °C.

#### Setup and data acquisition

Confocal laser scanning microscopy images were recorded with a LSM 710 (Carl Zeiss AG, Oberkochen, Germany) using a 633 nm laser and an oil immersion objective with 63 × magnification and NA = 1.4. Measurements were taken with the ZEN software (Carl Zeiss AG) using the following settings: an image size of 512 × 512 pixels, a scanning speed of 1 (pixel dwell time = 1.58 μs, scan time = 968.14 ms), no averaging, a bit depth of 16 bit, and a master gain of 800. The pinhole was adjusted to 1 Airy unit. A laser power of 15% was chosen. 10–20 cells were imaged for each condition.

For analysis, images were processed with imagej. Brightness and contrast were adjusted homogeneously for images showing the same cellular structures in order to compare cytotoxin‐treated cells to the respective control samples.

## Results and Discussion

### Resting receptors exhibit higher diffusion coefficients than activated MET

**Figure 1:**
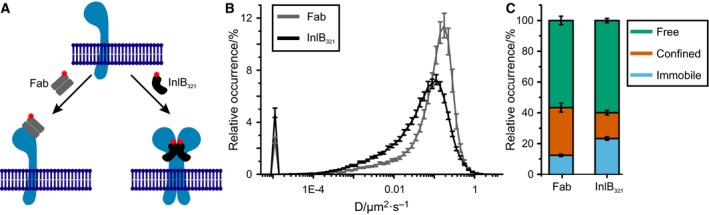
Diffusion dynamics of MET studied by uPAINT. (A) A monoclonal Fab fragment was used that binds but does not activate MET. InlB_321_ binds and activates the receptor. Both ligands were fluorescently labeled with ATTO 647N. (B) Distribution of diffusion coefficients (*N* = 60 cells) from uPAINT experiments for resting and InlB‐bound MET in living HeLa cells at 23 °C (SEMs are depicted as error bars). (C) Relative occurrences of immobile, confined, and freely diffusing particles of resting and InlB‐bound receptors.

We probed the diffusion dynamics of MET in living HeLa cells using uPAINT by applying two different ligands for MET, each carrying the fluorophore ATTO 647N. First, a monoclonal Fab fragment, which binds but does not activate MET, was deployed. Second, the bacterial protein fragment InlB_321_ was used (Fig. 1A). Cells were either investigated untreated or exposed to a cytotoxin affecting cell morphology and endocytosis pathways prior to analysis.

**Figure 2:**
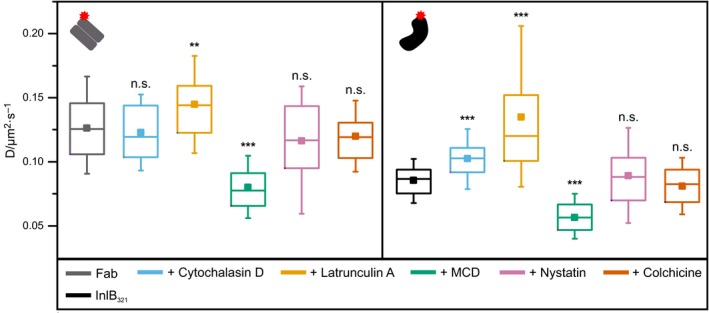
uPAINT mean diffusion coefficients of resting and InlB‐bound MET. Mean diffusion coefficients were determined for both ligand/MET complexes in living HeLa cells in combination with different cytotoxins. The box plot of diffusion coefficients (*N* = 60 cells) displays 5th percentile, 25th percentile, median (line), mean (square), 75th percentile, and 95th percentile. Results of two‐sample t‐tests comparing untreated cells with cells exposed to cytotoxins are depicted (*P* > 0.05 no significant difference between means (n.s.), *P* < 0.01 very significant difference (**), *P* < 0.001 highly significant difference (***)).

First, we studied MET in untreated cells incubated with 0.25 nm Fab or InlB_321_ at 23 °C and extracted the diffusion coefficients of the receptors. The distribution of diffusion coefficients showed that diffusion of InlB_321_/MET complexes is slower than that of resting MET (Fig. 1B). Analysis of diffusion types indicated a large immobile fraction for InlB‐bound receptors (Fig. 1C). While InlB_321_/MET had a mean diffusion coefficient of 0.085 ± 0.003 μm^2^·s^−1^, Fab/MET yielded an average coefficient of 0.126 ± 0.005 μm^2^·s^−1^ (Fig. 2, Table 1). Regardless of whether cells were cytotoxin‐treated or not, MET bound by Fab exhibited approximately 40% higher diffusion coefficients than receptors bound by InlB_321_ with a high significance (Fig. 2, Table 1, two‐sample *t*‐tests in Table 2).

**Table 1:** Diffusion coefficients of resting and InlB‐bound MET. The table lists the mean diffusion coefficients for 60 cells as well as mean values according to diffusion types with their respective SEMs for both ligand/MET complexes in combination with different cytotoxins

	*D* (μm^2^·s^−1^)			
	Mean	Immobile	Confined	Free
Fab	0.126 ± 0.005	0.0017 ± 0.0002	0.097 ± 0.006	0.162 ± 0.008
+ cytochalasin D	0.123 ± 0.005	0.0017 ± 0.0003	0.101 ± 0.006	0.160 ± 0.008
+ latrunculin A	0.145 ± 0.006	0.0017 ± 0.0004	0.109 ± 0.009	0.183 ± 0.010
+ MCD	0.080 ± 0.003	0.0018 ± 0.0002	0.066 ± 0.004	0.113 ± 0.006
+ nystatin	0.116 ± 0.006	0.0016 ± 0.0002	0.082 ± 0.006	0.183 ± 0.013
+ colchicine	0.120 ± 0.006	0.0017 ± 0.0003	0.096 ± 0.008	0.157 ± 0.010
At 32 °C	0.245 ± 0.005	0.0017 ± 0.0004	0.174 ± 0.013	0.306 ± 0.013
InlB_321_	0.085 ± 0.003	0.0017 ± 0.0001	0.051 ± 0.004	0.125 ± 0.005
+ cytochalasin D	0.102 ± 0.002	0.0018 ± 0.0002	0.056 ± 0.004	0.141 ± 0.006
+ latrunculin A	0.135 ± 0.008	0.0017 ± 0.0001	0.073 ± 0.005	0.185 ± 0.007
+ MCD	0.056 ± 0.002	0.0017 ± 0.0002	0.039 ± 0.004	0.090 ± 0.006
+ nystatin	0.089 ± 0.004	0.0016 ± 0.0002	0.058 ± 0.023	0.150 ± 0.013
+ colchicine	0.081 ± 0.002	0.0017 ± 0.0002	0.046 ± 0.004	0.116 ± 0.006
At 32 °C	0.194 ± 0.003	0.0019 ± 0.0002	0.095 ± 0.008	0.244 ± 0.008

**Table 2:** Two‐sample *t*‐tests comparing mean diffusion coefficients from MSD fits. *t*‐Values, degrees of freedom (df), *P*‐values, and levels of significance (LOS) from two‐sample t‐tests (α = 0.05) are listed. The sample of InlB_321_ + latrunculin A was not normally distributed according to the Kolmogorov–Smirnov test (α = 0.1). LOS are classified as follows: *P* > 0.05 no significant difference between means (n.s.), *P* < 0.01 very significant difference (**), and *P* < 0.001 highly significant difference (***)

Sample 1	Sample 2	*t*	df	*P*	LOS
Fab	Fab + cytochalasin D	0.7	118	0.5	n.s.
	Fab + latrunculin A	3.2	118	0.002	**
	Fab + MCD	10.7	118	3.4E‐19	***
	Fab + nystatin	1.7	118	0.09	n.s.
	Fab + colchicine	1.4	118	0.2	n.s.
	Fab at 32 °C	19.0	118	1E‐37	***
InlB_321_	InlB_321_ + cytochalasin D	6.0	118	3E‐08	***
	InlB_321_ + latrunculin A	6.4	118	3E‐09	***
	InlB_321_ + MCD	11.5	118	5E‐21	***
	InlB_321_ + nystatin	0.9	118	0.4	n.s.
	InlB_321_ + colchicine	1.6	118	0.1	n.s.
	InlB_321_ at 32 °C	30.1	118	3E‐57	***
Fab	InlB_321_	10.1	118	1E‐17	***
Fab + cytochalasin D	InlB_321_ + cytochalasin D	5.3	118	6E‐07	***
Fab + latrunculin A	InlB_321_ + latrunculin A	1.1	118	0.3	n.s.
Fab + MCD	InlB_321_ + MCD	8.1	118	6E‐13	***
Fab + nystatin	InlB_321_ + nystatin	4.6	118	1E‐05	***
Fab + colchicine	InlB_321_ + colchicine	10.4	118	3E‐18	***
Fab at 32 °C	InlB_321_ at 32 °C	8.5	118	7E‐14	***

In order to assess how temperature influences the diffusion coefficient of resting and InlB‐bound MET, we performed uPAINT experiments at 32 °C (c_ligand_ = 0.1 nm). As expected, the absolute diffusion coefficient increased according to the influence of temperature on membrane fluidity 38. The ratio between the diffusion coefficients of Fab/MET and InlB/MET remained constant (Table 1). All further experiments were performed at 23 °C.

**Figure 3:**
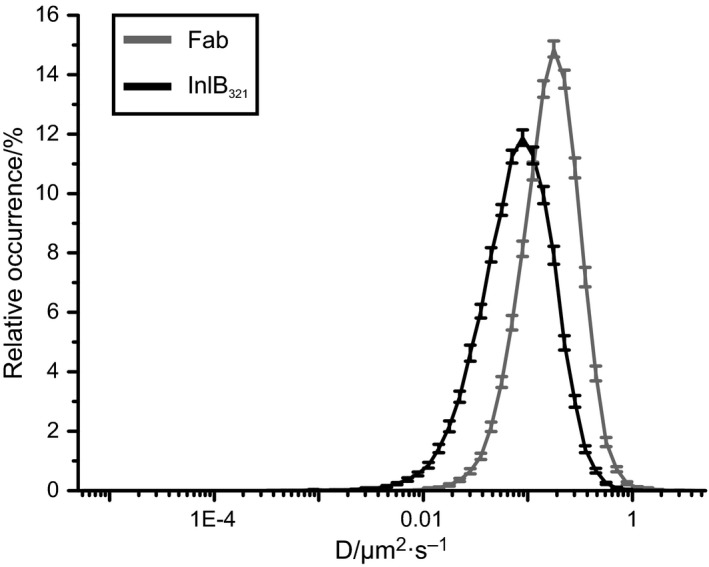
Relative occurrence of MET diffusion coefficients obtained by imFCS. A distribution of diffusion coefficients obtained by imFCS of InlB‐bound and resting MET at 23 °C was plotted (*N* = 60 cells). Distributions are shifted to higher diffusion coefficients compared to uPAINT data as imFCS does not recognize immobile particles. SEMs are depicted as error bars.

Diffusion coefficients of InlB‐bound and resting MET were also determined by imFCS (Fig. 3). Resting MET receptors were found to exhibit distinctly higher diffusion coefficients (0.197 ± 0.005 μm^2^·s^−1^) than InlB/MET (0.103 ± 0.003 μm^2^·s^−1^). The deviation from uPAINT is probably due to the fact that immobile particles cannot be detected by imFCS. Consistently, we found that diffusion coefficients of resting receptors are significantly higher than those of InlB_321_/MET complexes.

On the one hand, the observed ratio of diffusion coefficients between resting and InlB‐bound receptors supports the hypothesis that receptors are slowed by InlB binding possibly due to immobilization. On the other hand, this ratio might also be influenced by the increased occurrence of dimers in InlB‐activated cells 11. A study on epidermal growth factor receptor (EGFR) observed a factor of 2 between monomeric and dimeric diffusion coefficients by analyzing individual trajectories 39. Diffusion of the neurotrophin receptor tropomyosin‐related kinase A was also found to decelerate and to be more confined upon ligand binding due to receptor oligomerization 40.

### In comparison with other RTKs, ligand/MET complexes exhibit high diffusion coefficients

Diffusion coefficients of various RTKs span about three orders of magnitude (0.001–0.47 μm^2^·s^−1^) 39, 40, 41, 42, with MET belonging to the RTKs with high diffusion coefficients. For example, diffusion coefficients of 0.04 μm^2^·s^−1^ at 23 °C and of 0.001–0.01 μm^2^·s^−1^ at 37 °C were found for the insulin receptor using fluorescence photobleaching recovery. In the same study, diffusion coefficients of EGFR were found to be similar to those of the insulin receptor. The decrease in mobility with increasing temperature was explained by the more rapid aggregation and subsequent endocytosis of activated receptors at 37 °C 42. MET displayed a contrary behavior, with diffusion coefficients increasing with rising temperature for both resting and InlB‐bound receptors (Table 1). This effect could be due to decreasing membrane viscosity. A diffusion coefficient of 0.2 μm^2^·s^−1^ was observed for resting EGFR using imFCS 41. Examination of EGFR by single‐particle tracking yielded diffusion coefficients of 0.17 μm^2^·s^−1^ for monomers and 0.08 μm^2^·s^−1^ for preformed dimers. Upon binding of a ligand, diffusion coefficients were diminished to about 0.017 μm^2^·s^−1^ 39. Chung *et al*. discussed that this reduction in mobility is due to interaction with signaling proteins and preceding endocytosis. A similar mechanism can probably be assumed for MET which also exhibited decreased diffusion coefficients in the InlB‐bound state.

### Diffusion‐type analysis reveals a distinct immobile fraction for InlB_321_/MET complexes

We determined the diffusion types for MET from uPAINT data by MSD analysis. The mean diffusion coefficient (Table 1) and relative occurrence (Table 3) of each diffusion type were calculated. In the case of MET bound to Fab, the immobile population was smaller than the confined population (paired *t*‐test immobile vs. confined: *P* = 3E‐7). The population of freely diffusing complexes was dominant (paired *t*‐test free vs. confined: *P* = 3E‐5). InlB_321_‐bound MET displayed a small confined fraction, while the immobile population was significantly larger (paired t‐test immobile vs. confined: *P* = 0.005). The freely diffusing population was biggest (paired *t*‐test free vs. immobile: *P* = 5E‐26). Comparing InlB_321_ and Fab, the latter exhibited a distinctly smaller immobile population, while the confined population was significantly larger. The relative occurrences of free MET receptors showed no significant difference between the two ligands. However, the diffusion coefficients of freely and confined diffusing Fab/MET complexes were significantly higher than those of the corresponding populations of InlB_321_/MET. Observations of the same ligands at 32 °C revealed even higher diffusion coefficients for both mobile fractions. In both cases, temperature increase resulted in a decrease in the immobile fraction and a simultaneous increase in the mobile population (two‐sample *t*‐tests in Tables 4, 5, 6, 7, 8, 9).

**Table 3:** Relative occurrences of diffusion types of resting and InlB‐bound MET. The table lists the mean relative occurrences of diffusion types (*N* = 60 cells) with respective SEMs for both ligand/MET complexes in combination with different cytotoxins

	Relative occurrence (%)		
	Immobile	Confined	Free
Fab	12 ± 1	31 ± 3	57 ± 3
+ cytochalasin D	12 ± 1	38 ± 3	50 ± 3
+ latrunculin A	10 ± 1	31 ± 3	59 ± 3
+ MCD	16 ± 1	38 ± 3	47 ± 2
+ nystatin	24 ± 3	28 ± 3	48 ± 3
+ colchicine	13 ± 1	36 ± 3	52 ± 3
At 32 °C	7.2 ± 0.4	33 ± 2	60 ± 2
InlB_321_	23 ± 1	17 ± 2	60 ± 1
+ cytochalasin D	17 ± 1	19 ± 2	64 ± 2
+ latrunculin A	18 ± 1	18 ± 2	64 ± 2
+ MCD	26 ± 1	22 ± 2	51 ± 2
+ nystatin	25 ± 1	23 ± 3	52 ± 3
+ colchicine	23 ± 1	18 ± 2	59 ± 2
At 32 °C	10 ± 1	19 ± 2	70 ± 2

**Table 4:** Two‐sample *t*‐tests for diffusion coefficients from immobile subpopulations. *t*‐Values, degrees of freedom (df), p‐values, and levels of significance (LOS) from two‐sample *t*‐tests (α = 0.05) are listed. The sample of InlB_321_ at 32 °C was not normally distributed according to the Kolmogorov–Smirnov test (α = 0.1). LOS are classified as follows: *P* > 0.05 no significant difference between means (n.s.), *P* < 0.05 significant difference (*), and *P* < 0.01 very significant difference (**)

Sample 1	Sample 2	*t*	df	*P*	LOS
Fab	Fab + cytochalasin D	0.3	118	0.8	n.s.
	Fab + latrunculin A	0.9	118	0.4	n.s.
	Fab + MCD	0.9	118	0.4	n.s.
	Fab + nystatin	1.9	118	0.06	n.s.
	Fab + colchicine	0.1	118	0.9	n.s.
	Fab at 32 °C	0.4	117	0.7	n.s.
InlB_321_	InlB_321_ + cytochalasin D	2.3	115	0.02	*
	InlB_321_ + latrunculin A	1.2	115	0.2	n.s.
	InlB_321_ + MCD	1.7	115	0.1	n.s.
	InlB_321_ + nystatin	0.5	115	0.6	n.s.
	InlB_321_ + colchicine	0.7	115	0.5	n.s.
	InlB_321_ at 32 °C	2.9	114	0.004	**
Fab	InlB_321_	1.1	115	0.3	n.s.
Fab + cytochalasin D	InlB_321_ + cytochalasin D	0.8	118	0.4	n.s.
Fab + latrunculin A	InlB_321_ + latrunculin A	0.9	118	0.4	n.s.
Fab + MCD	InlB_321_ + MCD	0.4	118	0.7	n.s.
Fab + nystatin	InlB_321_ + nystatin	0.5	118	0.6	n.s.
Fab + colchicine	InlB_321_ + colchicine	0.3	118	0.8	n.s.
Fab at 32 °C	InlB_321_ at 32 °C	1.6	116	0.1	n.s.

**Table 5:** Two‐sample *t*‐tests for diffusion coefficients from confined subpopulations. *T*‐Values, degrees of freedom (df), *P*‐values, and levels of significance (LOS) from two‐sample *t*‐tests (α = 0.05) are listed. All samples were normally distributed according to the Kolmogorov–Smirnov test (α = 0.1). LOS are classified as follows: *P* > 0.05 no significant difference between means (n.s.), *P* < 0.01 very significant difference (**), and *P* < 0.001 highly significant difference (***)

Sample 1	Sample 2	*t*	df	*P*	LOS
Fab	Fab + cytochalasin D	0.6	112	0.5	n.s.
	Fab + latrunculin A	1.6	110	0.1	n.s.
	Fab + MCD	5.0	113	2E‐06	***
	Fab + nystatin	1.9	111	0.06	n.s.
	Fab + colchicine	0.2	112	0.9	n.s.
	Fab at 32 °C	7.9	112	2E‐12	***
InlB_321_	InlB_321_ + cytochalasin D	1.3	111	0.2	n.s.
	InlB_321_ + latrunculin A	3.2	109	0.002	**
	InlB_321_ + MCD	3.0	114	0.003	**
	InlB_321_ + nystatin	1.6	107	0.1	n.s.
	InlB_321_ + colchicine	1.1	113	0.3	n.s.
	InlB_321_ at 32 °C	5.5	109	2E‐07	***
Fab	InlB_321_	7.4	109	3E‐11	***
Fab + cytochalasin D	InlB_321_ + cytochalasin D	7.5	114	1E‐11	***
Fab + latrunculin A	InlB_321_ + latrunculin A	4.3	110	3E‐05	***
Fab + MCD	InlB_321_ + MCD	7.4	118	2E‐11	***
Fab + nystatin	InlB_321_ + nystatin	3.4	109	9E‐04	***
Fab + colchicine	InlB_321_ + colchicine	8.5	116	7E‐14	***
Fab at 32 °C	InlB_321_ at 32 °C	7.2	112	8E‐11	***

**Table 6:** Two‐sample *t*‐tests for diffusion coefficients from free subpopulations. *t*‐Values, degrees of freedom (df), *P*‐values, and levels of significance (LOS) from two‐sample *t*‐tests (α = 0.05) are listed. The samples of InlB_321_ + nystatin, InlB_321_ + Latrunculin A, and Fab + cytochalasin D were not normally distributed according to the Kolmogorov–Smirnov test (α = 0.1). LOS are classified as follows: *P* > 0.05 no significant difference between means (n.s.), *P* < 0.01 very significant difference (**), and *P* < 0.001 highly significant difference (***)

Sample 1	Sample 2	*t*	df	*P*	LOS
Fab	Fab + cytochalasin D	0.3	118	0.7	n.s.
	Fab + latrunculin A	2.7	118	0.007	**
	Fab + MCD	8.8	118	1E‐14	***
	Fab + nystatin	2.8	118	0.006	**
	Fab + colchicine	0.9	118	0.4	n.s.
	Fab at 32 °C	18.7	118	5E‐37	***
InlB_321_	InlB_321_ + cytochalasin D	4.4	115	3E‐05	***
	InlB_321_ + latrunculin A	5.7	115	1E‐07	***
	InlB_321_ + MCD	9.7	115	1E‐16	***
	InlB_321_ + nystatin	2.9	115	0.004	**
	InlB_321_ + colchicine	2.7	115	0.009	**
	InlB_321_ at 32 °C	23.3	115	2E‐45	***
Fab	InlB_321_	7.1	115	9E‐11	***
Fab + cytochalasin D	InlB_321_ + cytochalasin D	3.7	118	3E‐04	***
Fab + latrunculin A	InlB_321_ + latrunculin A	0.2	118	0.9	n.s.
Fab + MCD	InlB_321_ + MCD	5.6	118	2E‐07	***
Fab + nystatin	InlB_321_ + nystatin	3.2	118	0.002	**
Fab + colchicine	InlB_321_ + colchicine	9.6	118	2E‐16	***
Fab at 32 °C	InlB_321_ at 32 °C	8.2	118	4E‐13	***

**Table 7:** Two‐sample *t*‐tests for relative occurrences from immobile subpopulations. *t*‐Values, degrees of freedom (df), *P*‐values, and levels of significance (LOS) from two‐sample *t*‐tests (α = 0.05) are listed. The samples of Fab, Fab + nystatin, and Fab + cytochalasin D were not normally distributed according to the Kolmogorov–Smirnov test (α = 0.1). LOS are classified as follows: *P* > 0.05 no significant difference between means (n.s.), *P* < 0.01 very significant difference (**), and *P* < 0.001 highly significant difference (***)

Sample 1	Sample 2	*t*	df	*P*	LOS
Fab	Fab + cytochalasin D	0.5	118	0.6	n.s.
	Fab + latrunculin A	2.7	118	0.008	**
	Fab + MCD	3.4	118	0.001	**
	Fab + nystatin	4.3	118	4E‐05	***
	Fab + colchicine	0.4	118	0.7	n.s.
	Fab at 32 °C	5.9	118	4E‐08	***
InlB_321_	InlB_321_ + cytochalasin D	4.7	115	9E‐06	***
	InlB_321_ + latrunculin A	3.7	115	3E‐04	***
	InlB_321_ + MCD	1.9	115	0.06	n.s.
	InlB_321_ + nystatin	0.8	115	0.4	n.s.
	InlB_321_ + colchicine	0.5	115	0.6	n.s.
	InlB_321_ at 32 °C	11.0	115	1E‐19	***
Fab	InlB_321_	8.3	115	2E‐13	***
Fab + cytochalasin D	InlB_321_ + cytochalasin D	6.2	118	8E‐09	***
Fab + latrunculin A	InlB_321_ + latrunculin A	6.5	118	2E‐09	***
Fab + MCD	InlB_321_ + MCD	7.7	118	4E‐12	***
Fab + nystatin	InlB_321_ + nystatin	0.3	118	0.7	n.s.
Fab + colchicine	InlB_321_ + colchicine	9.5	118	3E‐16	***
Fab at 32 °C	InlB_321_ at 32 °C	4.8	118	5E‐06	***

**Table 8:** Two‐sample *t*‐tests for relative occurrences from confined subpopulations. *t*‐Values, degrees of freedom (df), *P*‐values, and levels of significance (LOS) from two‐sample *t*‐tests (α = 0.05) are listed. The samples of InlB_321_ + nystatin and Fab + cytochalasin D were not normally distributed according to the Kolmogorov–Smirnov test (α = 0.1). LOS are classified as follows: *P* > 0.05 no significant difference between means (n.s.), *P* < 0.05 significant difference (*), and *P* < 0.001 highly significant difference (***)

Sample 1	Sample 2	*t*	df	*P*	LOS
Fab	Fab + cytochalasin D	1.7	118	0.1	n.s.
	Fab + latrunculin A	0.1	118	0.9	n.s.
	Fab + MCD	1.7	118	0.09	n.s.
	Fab + nystatin	0.8	118	0.4	n.s.
	Fab + colchicine	1.1	118	0.3	n.s.
	Fab at 32 °C	0.4	118	0.7	n.s.
InlB_321_	InlB_321_ + cytochalasin D	0.8	115	0.4	n.s.
	InlB_321_ + latrunculin A	0.6	115	0.6	n.s.
	InlB_321_ + MCD	1.9	115	0.06	n.s.
	InlB_321_ + nystatin	2.0	115	0.05	*
	InlB_321_ + colchicine	0.4	115	0.7	n.s.
	InlB_321_ at 32 °C	0.9	115	0.4	n.s.
Fab	InlB_321_	4.3	115	4E‐05	***
Fab + cytochalasin D	InlB_321_ + cytochalasin D	5.5	118	2E‐07	***
Fab + latrunculin A	InlB_321_ + latrunculin A	4.0	118	1E‐04	***
Fab + MCD	InlB_321_ + MCD	4.3	118	3E‐05	***
Fab + nystatin	InlB_321_ + nystatin	1.1	118	0.3	n.s.
Fab + colchicine	InlB_321_ + colchicine	5.1	118	1E‐06	***
Fab at 32 °C	InlB_321_ at 32 °C	4.0	118	1E‐04	***

**Table 9:** Two‐sample *t*‐tests for relative occurrences from free subpopulations. T‐values, degrees of freedom (df), *P*‐values, and levels of significance (LOS) from two‐sample *t*‐tests (α = 0.05) are listed. The sample of Fab was not normally distributed according to the Kolmogorov–Smirnov test (α = 0.1). LOS are classified as follows: *P* > 0.05 no significant difference between means (n.s.), *P* < 0.05 significant difference (*), *P* < 0.01 very significant difference (**), and *P* < 0.001 highly significant difference (***)

Sample 1	Sample 2	*t*	df	*P*	LOS
Fab	Fab + cytochalasin D	1.6	118	0.1	n.s.
	Fab + latrunculin A	0.6	118	0.5	n.s.
	Fab + MCD	2.7	118	0.009	**
	Fab + nystatin	2.1	118	0.03	*
	Fab + colchicine	1.2	118	0.2	n.s.
	Fab at 32 °C	1.0	118	0.3	n.s.
InlB_321_	InlB_321_ + cytochalasin D	1.5	115	0.1	n.s.
	InlB_321_ + latrunculin A	1.8	115	0.07	n.s.
	InlB_321_ + MCD	3.3	115	0.001	**
	InlB_321_ + nystatin	2.5	115	0.01	*
	InlB_321_ + colchicine	0.2	115	0.9	n.s.
	InlB_321_ at 32 °C	3.8	115	2E‐04	***
Fab	InlB_321_	1.1	115	0.3	n.s.
Fab + cytochalasin D	InlB_321_ + cytochalasin D	4.0	118	1E‐04	***
Fab + latrunculin A	InlB_321_ + latrunculin A	1.6	118	0.1	n.s.
Fab + MCD	InlB_321_ + MCD	1.4	118	0.2	n.s.
Fab + nystatin	InlB_321_ + nystatin	0.9	118	0.4	n.s.
Fab + colchicine	InlB_321_ + colchicine	2.3	118	0.02	*
Fab at 32 °C	InlB_321_ at 32 °C	3.0	118	0.003	**

**Figure 4:**
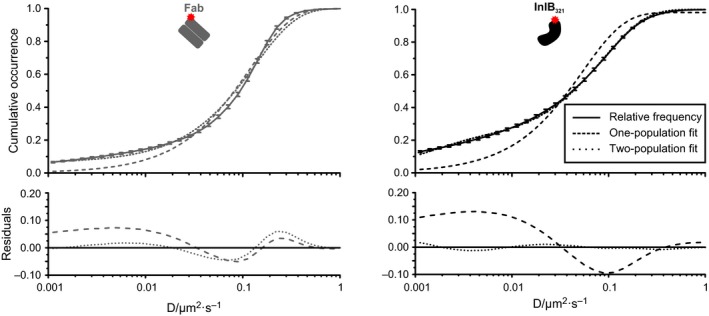
Cumulative occurrences of diffusion coefficients of resting and InlB‐bound MET. Diffusion coefficients and their respective SEMs in living HeLa cells (*N* = 60) at 23 °C are depicted. Plots were fitted with a one‐ and a two‐population function using Eqns (4) and (5). Residuals are shown below.

As a second approach, we used cumulative analysis 33 to determine populations of different diffusion coefficients. Cumulative occurrences were fitted with a one‐ and a two‐population function (Fig. 4, Table 10). An attempt to fit the plots with a three population function failed. Most likely, diffusion coefficients of the confined and free subpopulation are too similar and cannot be differentiated by cumulative analysis. In general, the two‐population fit converged better than the one‐population fit, indicating a mobile and an immobile fraction. However, the mobile fraction of InlB/MET decreases compared to resting receptors as indicated by the lower amplitude in the two‐population fit. This suggests that InlB_321_/MET is immobilized to a higher extent than MET bound by Fab.

**Table 10:** Diffusion coefficients and amplitudes from the fits of cumulative occurrences. Results with respective SEMs are shown for one‐ and two‐population fits (Eqns (4) and (5)) of the mean cumulative occurrences of diffusion coefficients obtained by uPAINT at 23 and 32 °C (*N* = 60 cells). Amplitudes signify the relative proportion of particles whose motion can be described accurately by the respective diffusion coefficient

	One population		Two populations		
	A_1_	D_1_/μm²·s^−1^	A_1_	D_1_/μm²·s^−1^	D_2_/μm²·s^−1^
Fab	1.00 ± 0.01	0.119 ± 0.005	0.94 ± 0.01	0.134 ± 0.004	4E‐4 ± 9E‐4
+ cytochalasin D	1.00 ± 0.01	0.119 ± 0.005	0.95 ± 0.01	0.131 ± 0.004	2E‐4 ± 1E‐3
+ latrunculin A	1.00 ± 0.01	0.143 ± 0.005	0.96 ± 0.01	0.153 ± 0.005	3E‐6 ± 0
+ MCD	1.00 ± 0.01	0.067 ± 0.003	0.92 ± 0.01	0.080 ± 0.001	5E‐4 ± 2E‐4
+ nystatin	0.98 ± 0.02	0.077 ± 0.008	0.79 ± 0.01	0.135 ± 0.002	0.001 ± 9E‐5
+ colchicine	1.00 ± 0.01	0.116 ± 0.005	0.94 ± 0.01	0.130 ± 0.004	2E‐4 ± 2E‐3
At 32 °C	1.01 ± 0.01	0.259 ± 0.011	0.97 ± 0.01	0.269 ± 0.011	1E‐5 ± 0
InlB_321_	0.98 ± 0.02	0.054 ± 0.004	0.81 ± 0.01	0.089 ± 0.001	0.0014 ± 8E‐5
+ cytochalasin D	0.99 ± 0.01	0.076 ± 0.004	0.87 ± 0.01	0.103 ± 0.001	0.0013 ± 7E‐5
+ latrunculin A	0.98 ± 0.01	0.091 ± 0.006	0.86 ± 0.01	0.130 ± 0.001	0.0016 ± 1E‐4
+ MCD	0.98 ± 0.01	0.036 ± 0.003	0.81 ± 0.01	0.058 ± 0.001	0.0013 ± 8E‐5
+ nystatin	0.97 ± 0.02	0.049 ± 0.005	0.76 ± 0.01	0.100 ± 0.001	0.0014 ± 8E‐5
+ colchicine	0.98 ± 0.02	0.053 ± 0.004	0.82 ± 0.01	0.084 ± 0.001	0.0014 ± 7E‐5
At 32 °C	1.00 ± 0.01	0.176 ± 0.008	0.92 ± 0.01	0.205 ± 0.004	0.0013 ± 4E‐4

**Figure 5:**
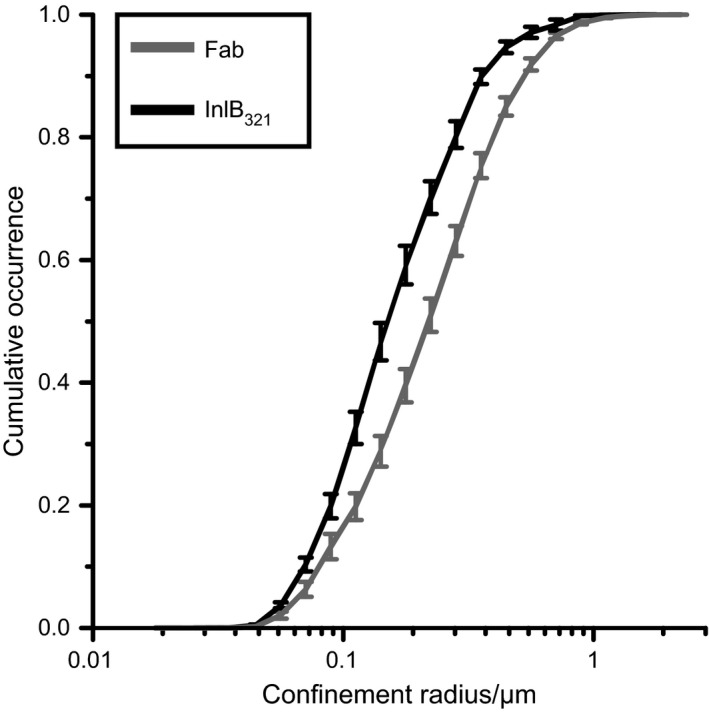
Cumulative occurrences of confinement radii for resting and InlB‐bound MET. Cumulative occurrences of confinement radii for InlB_321_/MET and Fab/MET obtained from the uPAINT data are shown (*N* = 60 cells). Error bars represent the SEMs of individual data points.

Confinement radii were determined for both ligand/MET complexes (Fig. 5). Particles can either be confined by actin or by nanodomains, for example, sterol‐rich regions 20. Domains confined by actin are thought to have a confinement radius of 0.2–0.6 μm 20. Diffusion hampered by nanodomains exhibits even smaller radii, ranging from 10 nm up to 200 nm 14. Confinement radii of untreated cells amounted to 0.276 ± 0.021 μm for Fab/MET and 0.188 ± 0.022 μm for InlB_321_/MET. At 32 °C, confinement radii increased for both ligands (0.376 ± 0.027 μm for Fab/MET, 0.249 ± 0.022 μm for InlB_321_/MET). These values are in the range of both the domain size of confinement by actin and of nanodomains.

Diffusion coefficients, diffusion types, and confinement radii suggest that resting MET diffuses more freely than MET bound by InlB_321_. This may support the hypothesis of immobilization prior to endocytosis as also discussed by Chung *et al*. for EGFR 39. Lower diffusion coefficients and a higher immobile population of InlB_321_‐bound receptors might be due to the formation of a signaling complex comprising several proteins, such as growth factor receptor‐bound protein 2 (Grb2) or Grb2‐associated binding protein 1 (Gab1) 3, 43, 44, 45 and coreceptors such as CD44v6 46.

### Perturbing actin polymerization and depletion of cholesterol influence MET diffusion

**Figure 6:**
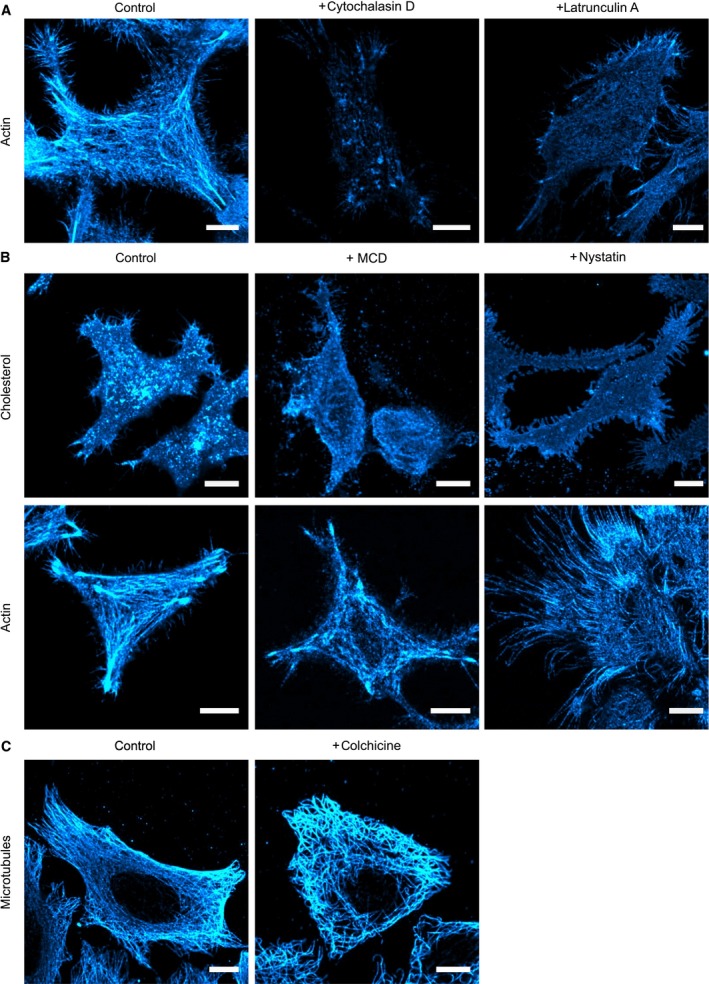
Effects of cytotoxins on different cellular structures visualized by confocal microscopy. CLSM images of effector‐treated HeLa cells as well as of untreated control samples are represented. After fixation, (A + B) actin was stained with phalloidin‐Alexa Fluor 647, (B) cholesterol‐rich regions were stained with cholera toxin subunit B‐Alexa Fluor 647, and (C) microtubules were immunostained with Alexa Fluor 647. All images were adjusted to the same intensity and contrast as their respective controls in ImageJ (scale bars 10 μm).

The effects of actin, cholesterol, and microtubules on plasma membrane mobility of MET were studied by uPAINT in living cells after exposure to selected cytotoxins. In cells treated with cytochalasin D and latrunculin A, which affect actin polymerization, increased diffusion coefficients were found for InlB_321_/MET. While cytochalasin D caused an increase in the diffusion coefficient by approximately 20%, latrunculin A led to an increase of about 60%. In the case of resting MET bound by Fab, treatment with cytochalasin D did not result in a significant change in the diffusion coefficient, and latrunculin A induced only a slight increase in the diffusion coefficient (Table 1, Fig. 2) resulting in an insignificant difference between diffusion in resting and InlB‐treated cells (two‐sample *t*‐tests in Table 2). Latrunculin A disrupts the actin cytoskeleton very effectively (Fig. 6). Actin is suspected to play an essential role in the immobilization of activated MET 46. If the actin cytoskeleton is destroyed so that immobilization and confinement of proteins is significantly decreased, the difference in diffusion between resting and InlB‐bound receptors is diminished. This hypothesis is in accordance with Chung *et al*. who suggest that latrunculin treatment lowers the dimerization frequency by increasing the diffusible area, effectively reducing receptor density 39.

In order to investigate the influence of cholesterol, cells were exposed to the cholesterol‐depleting cytotoxins MCD and nystatin. While MCD among other effects mainly prevents clathrin‐dependent endocytosis, nystatin inhibits caveolae‐dependent internalization 16, 18. Addition of MCD decreased the diffusion coefficients of resting as well as of InlB_321_‐bound receptors with a high significance. Mean diffusion coefficients suggest that treatment with nystatin had no obvious effect on the diffusion of InlB_321_/MET complexes (Table 1, Fig. 2, two‐sample *t*‐tests in Table 2).

Cells were exposed to colchicine to study the influence of microtubules on MET diffusion. Neither the diffusion coefficients of Fab/MET nor of InlB_321_/MET were influenced by colchicine treatment ruling out a major role of microtubules in MET diffusion (Table1, Fig. 2, two‐sample *t*‐tests in Table 2).

In addition to calculating overall averaged diffusion coefficients, we also determined the diffusion coefficients of immobile, confined, and freely moving MET populations and their share of all detected particles by MSD analysis for cytotoxin‐treated cells (Tables 1 and 3, two‐sample *t*‐tests in Tables 4, 5, 6, 7, 8, 9). Both actin‐targeting cytotoxins significantly decreased the immobile population of InlB_321_/MET. Additionally, latrunculin A treatment increased the diffusion coefficients of both mobile fractions, while exposure to cytochalasin D only caused a rise in the diffusion coefficient of free receptor. For Fab, there was no significant change in relative occurrences of the populations and their diffusion coefficients after exposure to cytochalasin D. Latrunculin A addition resulted in a slight decrease in the immobile population and a faintly higher diffusion coefficient of freely diffusing particles. Next, cholesterol‐depleting and endocytosis‐inhibiting drugs were tested. Treatment with MCD caused a decrease in the free fraction for both ligands, while the diffusion coefficients of both mobile populations declined sharply. Nystatin exposure resulted in an increase in the diffusion coefficients of the free populations, which could be a result of nystatin rupturing the actin cytoskeleton (Fig. 6). While effects on the fractions of subpopulations of InlB‐bound receptors were negligible, a highly significant increase in the immobile fraction of resting MET was observed. Exposure to the microtubule‐targeting cytotoxin colchicine did not result in altered diffusion coefficients of subpopulations compared to untreated cells.

Cumulative analysis of diffusion coefficients in cytotoxin‐treated cells (Table 10) showed an increased accuracy of the one‐population fit for treatment groups exhibiting small immobile populations and high mean diffusion coefficients (cytochalasin D, latrunculin A) in the MSD analysis (Tables 1 and 3). For groups displaying a distinct immobile population and low mean diffusion coefficients (MCD), the two‐population fit converged much better. Thus, cumulative analysis of subpopulations support the result obtained by MSD analysis.

The effects of the applied cytotoxins on cell integrity were also examined by confocal microscopy (Table 11, Fig. 6). Treatment with cytochalasin D and latrunculin A led to torn and dissolved actin filaments. MCD and nystatin resulted in a decreased staining of cholesterol‐rich domains, and actin filaments became porous and frayed at the cell edges. Colchicine caused the microtubules to loosen and curl.

**Table 11:** Effects of applied cytotoxins. The table lists the effects of the cytotoxins applied to living HeLa cells as well as references where these effects were observed. Impacts on cellular structures marked with (x) were observed in CLSM images (see Fig. 6) but are not mentioned in the literature

	Damage to actin	Damage to microtubules	Cholesterol depletion	Inhibition of clathrin‐mediated endocytosis	Inhibition of caveolae‐dependent endocytosis	References
Cytochalasin D	x					Cooper 21
Latrunculin A	x					Yarmola *et al*. 22
MCD	(x)		x	x	x	Yancey *et al*. 15 Cubi *et al*. 17 Rodal *et al*. 16
Nystatin	(x)		x		x	Ros‐Baro *et al*. 18
Colchicine		x				Ravelli *et al*. 24

#### Immobilization of InlB_321_‐bound receptors

The results obtained after treatment with the actin‐targeting drugs cytochalasin D and latrunculin A suggest that immobilization of InlB_321_/MET involves an interaction with the actin cytoskeleton. It was previously supposed that the MET coreceptor CD44v6 interacts with actin via ezrin 46. At the same time, both drugs had only a small or no effect on resting receptors, supporting the hypothesis that InlB‐bound MET is immobilized preferentially as a first step of endocytosis. Presumably, this difference is due to the fact that InlB‐bound MET receptors are bound to actin by the abovementioned proteins of the signaling cascade 46, while resting MET seems to be barely affected.

#### Treatment with MCD and nystatin influences MET diffusion dynamics

In cells treated with MCD, we observed lower mean diffusion coefficients for both resting and InlB‐bound MET. Fractions of immobile receptors were increased, diffusion coefficients for the mobile fractions were decreased (Tables 1 and 3), and a better convergence of the two‐population fit in cumulative diffusion coefficient plots was obtained (Table 10). These results suggest that clathrin‐dependent endocytosis might play a role in the internalization of active MET, which would be in accordance with the results published by Li *et al*. 8. As MCD causes clathrin‐coated pits to stay fixed at the cell surface 15, immobilized receptors would not vanish from the membrane through internalization. However, caveolae‐dependent internalization of MET cannot be ruled out 16.

An alternative drug that affects cholesterol levels in membranes is nystatin, which has different coeffects including the inhibition of caveolae‐dependent endocytosis 18. In cells treated with nystatin, we observed only a small effect on the relative occurrence of subpopulations of InlB/MET, while the immobile fraction of Fab/MET significantly increased (Table 3). We suspect that resting receptors are immobilized, possibly for subsequent internalization, and that receptor recycling of inactive receptors might be caveolae dependent. For EGFR, it was shown that resting receptors are enriched in caveolae and that EGFR exits from caveolae upon activation and is internalized by clathrin‐coated pits 47. Future studies have to elucidate whether a similar mechanism holds for MET.

#### Microtubules do not influence the diffusion of ligand/MET complexes

As microtubules play a role in the uptake of *L. monocytogenes* 48, this cytoskeletal structure was targeted with colchicine. The results of this study showed no effect of colchicine, neither on the diffusion coefficients nor on the proportions of diffusion types. Microtubules did not hinder the diffusion of MET within the cell membrane or harbor binding sites for activated receptors.

## Conclusion

On the basis of this study and by considering previously published data, we come to the following conclusion for the dynamics of resting and InlB‐bound MET receptors. Diffusing receptors can be bound by the bacterial ligand InlB_321_. InlB‐bound receptors exhibit lower diffusion coefficients than resting ones. Previous studies have shown that upon binding by InlB, the dimer population increases 11 and 2 : 2 ligand/MET complexes form 49. These complexes are bound by proteins and cofactors 46. Our uPAINT data reveal lower diffusion coefficients and an increased immobile fraction for InlB_321_/MET. Immobilization presumably occurs along the actin cytoskeleton which can be reduced by exposure to actin‐targeting cytotoxins, such as cytochalasin D or latrunculin A. An earlier study reported that InlB‐activated MET receptors are internalized via clathrin‐coated pits 8. Addition of MCD inhibiting mainly clathrin‐mediated endocytosis but also affecting caveolae‐dependent internalization increased the immobile fraction of InlB_321_/MET complexes in our study.

It remains to be clarified whether differentiation between endocytosis pathways is possibly due to sorting of MET receptors to specific membrane domains, for example, by the actin cytoskeleton. Assays for endocytosis may provide evidence for the proposed endocytosis pathways. The presented results deliver comprehensive details on the diffusion behavior of MET within the cell membrane. Future studies may address how MET receptor complexes initiate intracellular signaling cascades, for example, via Grb2 or Gab1.

## Author contributions

MSD, MH, and HHN designed the study and supervised the experiments. MLH, MSD, and MH wrote the manuscript. MLH, PY, WMB, TM, CK, and MSD performed the experiments. PY and MLH wrote software code for data analysis. MLH, MSD, and MH analyzed data and designed figures.
